# Tumour growth environment modulates Chk1 signalling pathways and Chk1 inhibitor sensitivity

**DOI:** 10.1038/srep35874

**Published:** 2016-10-24

**Authors:** Andrew J. Massey

**Affiliations:** 1 Vernalis Research, Granta Park, Cambridge, CB21 6GB, UK.

## Abstract

Clinical development of Chk1 inhibitors is currently focussed on evaluating activity as monotherapy and as potentiators of chemotherapy. To aid translation of pre-clinical studies, we sought to understand the effects of the tumour growth environment on Chk1 signalling and sensitivity to small molecule Chk1 inhibition. Spheroid culture altered Chk1 signalling to a more xenograft like state but decreased sensitivity to Chk1 inhibition. Growth in low serum did not alter DDR signalling but increased the sensitivity of A2058 and U2OS tumour cells to Chk1 inhibition. An analysis of the expression levels of replication associated proteins identified a correlation between Cdc6 and pChk1 (S296) as well as total Chk1 in xenograft derived samples and between Cdc6 and total Chk1 in anchorage-dependent growth derived protein samples. No apparent correlation between Chk1 or Cdc6 expression and sensitivity to Chk1 inhibition *in vitro* was observed. A database analysis revealed upregulation of *CDC6* mRNA expression in tumour compared to normal tissue and a correlation between *CDC6* and *CHEK1* mRNA expression in human cancers. We suggest that Cdc6 overexpression in human tumours requires a concomitant increase in Chk1 to counterbalance the deleterious effects of origin hyperactivation-induced DNA damage.

Increased replication stress, due to oncogene activation and/or inactivation of tumour suppressor genes, is a common feature of many cancers. Replication stress can arise through numerous mechanisms including deregulated origin firing, increased DNA damage through increased ROS production, collision of active replication forks with the transcription factories and the chromatin context of replicating DNA. Loss of the controls restricting the onset of S-phase results in an unscheduled and uncoordinated replication burst that is not matched by the supply of components necessary for replication fork progression resulting in replication fork stalling, fork collapse and the generation of DNA strand breaks^1^,^2^,^3^.

Cellular DNA is subjected to daily damage form a range of intra- and extra-cellular processes. A series of sophisticated cell cycle checkpoint and DNA repair pathways (collectively termed the DNA damage response (DDR) of which ATR and Chk1 are key components) have evolved to help cells cope with this^4^,^5^,^6^. Binding of RPA to the extensive regions of ssDNA generated following replication fork stalling recruits ATR. Following activation by TOPBP1, ATR phosphorylates Chk1 on S317 and S345^7^,^8^ leading to Chk1 *cis*-autophosphorylation on S296^9^. Chk1 kinase activation leads to cell cycle arrest (via the phosphorylation dependent degradation of Cdc25 phosphatases), fork stabilisation and inhibition of cleavage by the Mus81-Eme1-Mre11 nucleases, activation of homologous recombination repair and inhibition of new origin firing^10^. Preventing fork cleavage allows replication restart once the damage has either been repaired or bypassed.

The major focus of the clinical development of Chk1 inhibitors has been as chemopotentiators of standard-of-care cytotoxic chemotherapy drugs (including gemcitabine, pemetrexed and cisplatin^11^). Inhibiting Chk1 thereby supressing the replication stress response has the capacity to elevate DNA damage induced by replicative stress and tumour cell death. Single agent Chk1 inhibitor activity has been observed in a range of cancer cell lines^12^,^13^,^14^,^15^,^16^,^17^ and genetically engineered tumour models^18^,^19^. These are characterised as containing defects in DNA repair pathways or elevated replicative stress. A series of Phase I and II trials are currently underway testing this hypothesis. The majority of pre-clinical, single-agent Chk1 inhibitor studies have utilised established cancer cell lines to identify sensitive models. Understanding the effects of the tumour growth environment on Chk1 signalling and sensitivity to small molecule Chk1 inhibition as well as differences in DDR pathway signalling between the *in vitro* and *in vivo* context is important for guiding the clinical evaluation of Chk1 inhibitors.

## Results

### Modulation of the cell culture environment to more closely mimic *in vivo* growth conditions does not alter DDR signalling in tumour cells growing anchorage dependently

**Figure 1:**
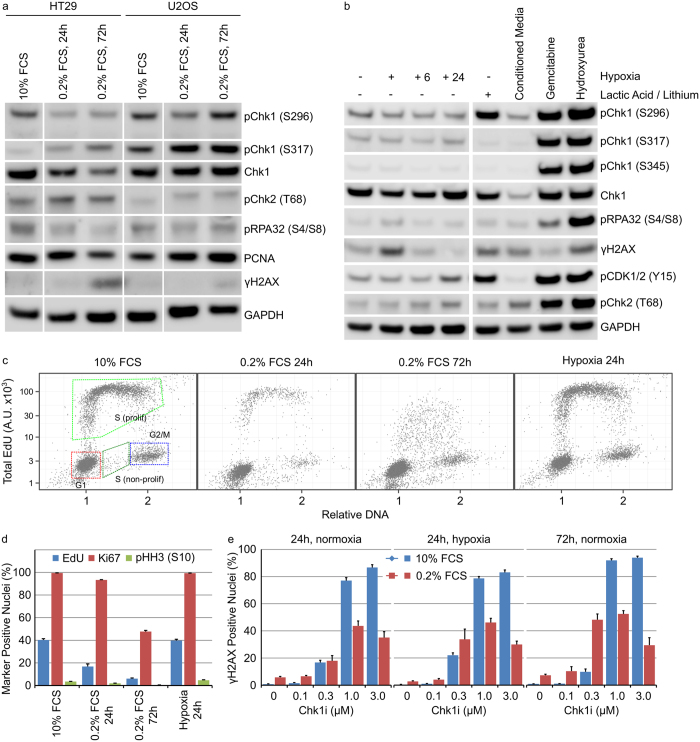
Modulation of the cell culture environment does not dramatically alter DDR signalling in tumour cells growing anchorage dependently. (**a**) HT29 or U2OS cells were grown in low serum for 24 or 72 hours. (**b**) HT29 cells, in 10% FCS containing media, were grown in: hypoxia for 24 hours with or without re-oxygenation for 6 (+6) or 24 (+24) hours or normoxia with 25 mM lactic acid, conditioned media, 50 nM gemcitabine or 2.5 mM hydroxyurea for 24 hours. Cell lysates were immunoblotted using the indicated antibodies. (**c**) The cell cycle distribution of HT29 cells cultured as described was determined by single cell immunofluorescent analysis (n = 4, mean ± SD). (**d**) The fraction of HT29 cells staining positive for EdU, Ki67 or pHH3 (S10) was determined by single cell immunofluorescent analysis (n = 4, mean ± SD). (**e**) HT29 cells were grown under the indicated culture conditions in combination with Chk1i for 24 or 72 hours. The number of γH2AX positive nuclei was determined by single cell immunofluorescent imaging (n = 4, mean ± SD).

Human tumours exist as complex organs growing in three-dimensions with complex cell-cell interactions and gradients of pH, nutrients and oxygen. Removal of cellular nutrients, such as glutamine or glucose, reduced pChk1 (S296) and total Chk1 protein levels in HT29 and U2OS cells that correlated with decreased DNA synthesis (Massey, manuscript submitted). We therefore sought to evaluate the effect of altered cell culture conditions on Chk1 signalling. Growth of HT29 or U2OS cells in very low (0.2%) FCS for 24 or 72 hours resulted in a decreased phosphorylation of Chk1 on serine 296 but increased phosphorylation on serine 317 (Fig. 1a). The anti-metabolites gemcitabine and hydroxyurea have previously been demonstrated to strongly activate DDR signalling in HT29 cells. Treatment of HT29 cells with 50 nM gemcitabine or 2.5 mM hydroxyurea induced DNA damage and strongly activated the DDR signalling response. Culturing HT29 cells in hypoxia (0.1% O_2_) for 24 hours coupled with subsequent re-oxygenation for 6 or 24 hours had no effect on DDR signalling (Fig. 1b). Addition of cell culture media conditioned for 5 days to HT29 cells decreased pChk1 (S296) and Chk1 protein levels whilst 25 mM lactic acid increased Chk1 autophosphorylation. However, the lithium salt of lactic acid was used and previous work has demonstrated a role for lithium in the activation of Chk1^20^. Treatment of HT29 or U2OS cells with 25 mM sodium lactate had no effect on Chk1 phosphorylation (Supplementary Fig. S1). Growth in very low serum but not hypoxia resulted in decreased DNA replication, decreased mitosis and an increase in quiescent HT29 cancer cells (Fig. 1c,d). Decreased DNA replication in low serum correlated with a decrease in the fraction of HT29 cells harbouring DNA damage (γH2AX-positive nuclei) following Chk1i treatment (Fig. 1e). We determined the effect of growth under hypoxic conditions on the sensitivity of HT29 cells to the Chk1 inhibitor V158411 (Chk1i)^21^. Hypoxia had no effect on the sensitivity of HT29 cells to Chk1i (GI_50_ 1.0 ± 0.29 μM) compared to HT29 cells grown in normoxia (GI_50_ 0.83 ± 0.25 μM, Supplementary Fig. S1).

### Growth in low FCS increases the susceptibility of tumour cells to inhibition of Chk1

**Figure 2:**
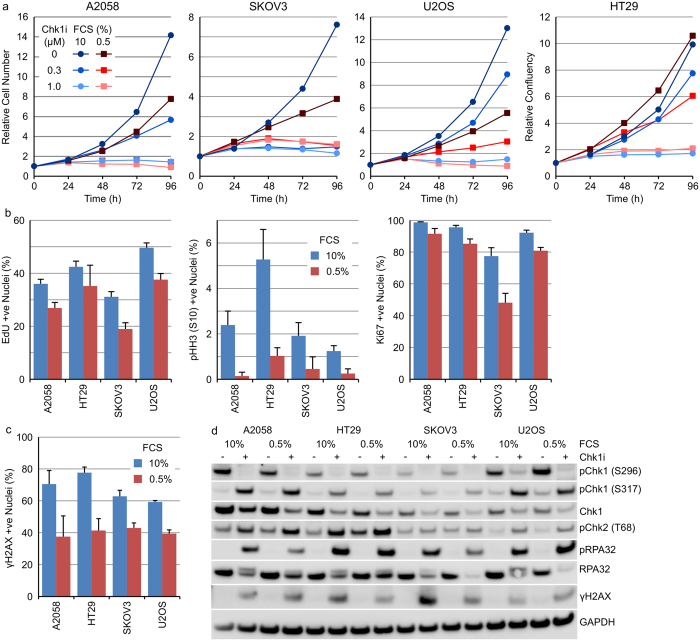
Growth in low FCS increases the sensitivity of A2058 and U2OS but not HT29 or SKOV3 tumour cells to Chk1 inhibition. (**a**) Tumour cell lines growing in 10% or 0.5% FCS were treated with 0–1 μM Chk1i for 96 hours. Cell number or cellular confluency was determined by repeated live cell imaging (n = 6, mean). (**b**) Tumour cell lines were grown in 10% or 0.5% FCS for 48 hours and the number of Ki67, pHH3 (S10) or EdU positive nuclei determined by single cell immunofluorescent imaging (n = 4, mean ± SD). (**c**) Tumour cell lines were grown in 10% or 0.5% FCS for 48 hours then treated with 1 μM Chk1i for a further 24 hours. The number of γH2AX positive nuclei determined by single cell immunofluorescent imaging (n = 4, mean ± SD). (**d**) Tumour cell lines growing in 10% or 0.5% FCS were treated with 0 or 1 μM Chk1i for 24 hours. Cell lysates were immunoblotted using the indicated antibodies.

Inhibiting tumour cell growth dramatically decreases DNA damage and replication stress induced by Chk1 inhibition. To further probe the effect of reduced growth factor supply on Chk1 inhibitor sensitivity, we determined the sensitivity of A2058, HT29, SKOV3 or U2OS cells to Chk1i at a concentration of FCS (0.5%) that resulted in reduced (but not complete inhibition of) cellular growth. To understand the effects on cell proliferation, daily live cell imaging was utilised. Reduced FCS increased the sensitivity of A2058 and U2OS cells to Chk1i whilst SKOV3 and HT29 cells displayed the same level of sensitivity as cells grown in 10% FCS (Fig. 2a and Supplementary Fig. S2). These observations were confirmed using SRB after 96 hours treatment with Chk1i (Supplementary Fig. S2). Culturing the cells for 48 hours in 0.5% FCS prior to treatment with 1 μM Chk1i resulted in increased sensitivity of A2058 and U2OS cells to Chk1i compared to cells grown in 10% FCS (Supplementary Fig. S2). Growth in low serum induced a small reduction in the fraction of cells actively synthesising DNA and decreased mitosis without a notable induction of quiescence except in SKOV3 cells where reduced serum increased the fraction of Ki67 negative cells 2.3-fold (Fig. 2b). A small reduction of 1.5- to 1.9-fold in the percentage of cells exhibiting γH2AX staining following Chk1i treatment was observed in cells growing in low FCS (Fig. 2c). No discernible differences in phosphorylation of proteins involved in the DDR pathway was observed following Chk1i treatment of A2058, HT29, SKOV3 or U2OS cells growing in 10% or 0.5% FCS (Fig. 2d).

### Anchorage-independent growth alters DDR signalling and decreases tumour cell sensitivity to Chk1i

Cancer cells growing attached to a plastic substrate in two-dimensions (2D) are oversupplied with growth factors and nutrients and with a relatively rapid and unlimited proliferative capacity have been the mainstay of cancer drug discovery. In reality, human tumours exist as complex organs growing in three-dimensions with complex cell-cell interactions and gradients of pH, nutrients and oxygen. These more complex microenvironments involving cell-cell and cell-matrix interactions as well as biochemical signals and nutrient gradients modulate cell signalling pathways and tumour cell growth. Three-dimensional cell culture models (such as multicellular tumour spheroids) allow the generation of concentration gradients of oxygen, growth factors and nutrients as well as cell-cell and cell-matrix interactions and serve as a useful intermediate model between 2D culture and *in vivo* xenograft models^22^,^23^.

**Figure 3:**
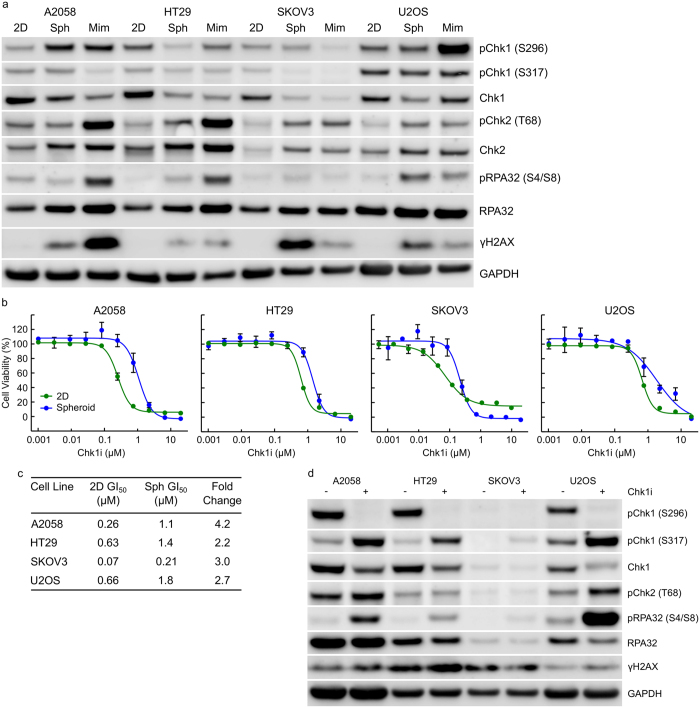
Anchorage-independent growth alters DDR signalling and decreases tumour cell sensitivity to Chk1i. (**a**) Tumour cell lines were grown anchorage-dependently (2D), as multi-cellular tumour spheroids (Sph) for 10 days or in mimetix 3D scaffold (Mim) for 14 days. Cell lysates were immunoblotted with the indicated antibodies. (**b**) Tumour cells growing anchorage-dependently (2D) or as multi-cellular tumour spheroids were treated with increasing concentrations of Chk1i for 3 days (2D) or 7 days (spheroids). Cell viability was determined using SRB or CellTiter-Glo (n ≥ 3, mean ± SD). (**c**) Concentrations of Chk1i that inhibited cell growth by 50% (GI_50_) were calculated from B. (**d**) Multi-cellular tumour spheroids were treated with 0 or 1 μM Chk1i for 24 hours. Cell lysates were immunoblotted with the indicated antibodies.

We therefore evaluated the effect of growth as tumour spheroids or in a mimetix scaffold on Chk1 kinase activity in A2058, HT29, SKOV3 or U2OS cells. The *cis*-autophosphorylation site on Chk1 (S296) serves as a useful biomarker of Chk1 kinase activity. Anchorage-independent growth altered Chk1 kinase activity in tumour cell lines compared to anchorage-dependent growth (Fig. 3a and Supplementary Fig. S3). Growth of A2058, SKOV3 or U2OS cells anchorage-independently or HT29 cells in mimetix increased pChk1 (S296) whilst HT29 spheroids had unchanged levels of pChk1 (S296). We subsequently determined the effect of anchorage independent growth on DDR pathway activation using pChk1 (S317), pChk2 (T68) and pRPA32 (S4/S8) as markers of ATR, ATM and DNA-PKcs activation respectively. γH2AX was utilised as a marker of DNA damage (see Supplementary Table S1 for details of protein function). Anchorage-independent growth resulted in increased DNA-PKcs activation and γH2AX under spheroid and mimetix growth conditions (Fig. 3a and Supplementary Fig. S3). We observed minimal increased pChk1 (S317) even in those samples with increased pChk1 (S296).

The effect of anchorage-independent growth on the sensitivity of A2058, HT29, SKOV3 or U2OS cells to Chk1i was determined. Anchorage-independent growth rendered A2058, HT29, SKOV3 or U2OS cells 4.2-, 2.2-, 3.0- and 2.7-fold more resistant to growth inhibition by Chk1i (Fig. 3b,c) compared to the same cell line growing anchorage-dependently. Treatment of multicellular tumour spheroids with 1 μM Chk1i inhibited Chk1 autophosphorylation (pS296) and DNA damage in the tumour spheroids (Fig. 3d) suggesting reasonable penetration of Chk1i into the spheroid.

### A comparative analysis of Chk1 signalling identifies differences between tumour cells growing anchorage-dependently and as xenografts in *nu/nu* mice

**Figure 4:**
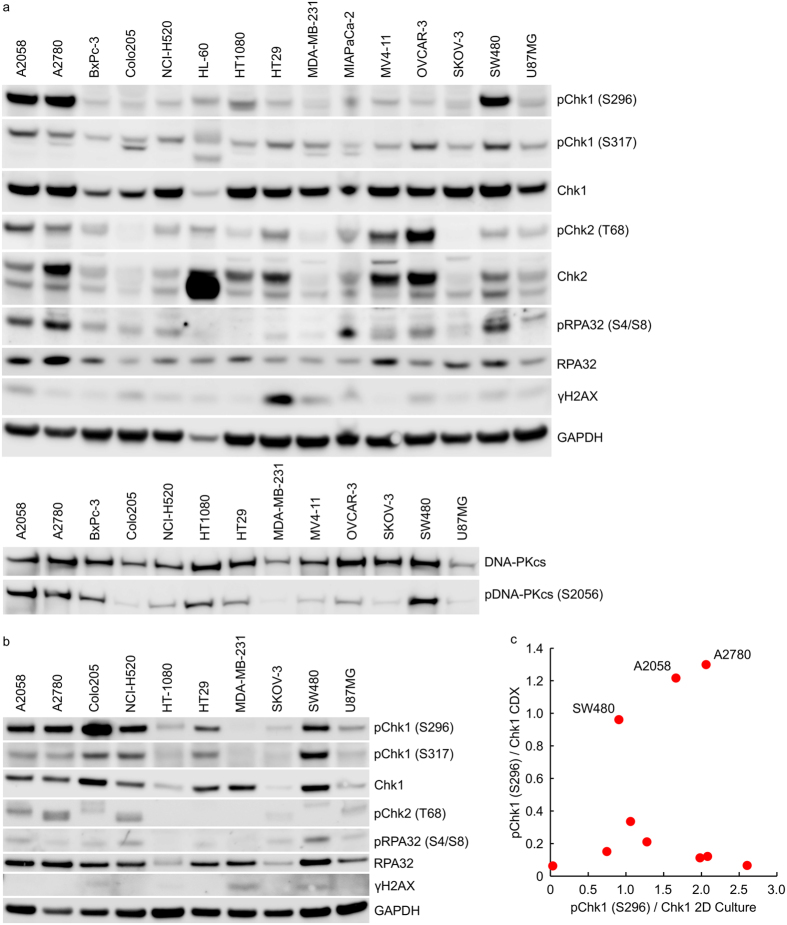
Comparative analysis of Chk1 signalling in tumour cells growing in anchorage-dependent cell culture and as xenografts in *nu/nu* mice. Cell lysates prepared from (**a**) cell line derived tumour xenografts or (**b**) anchorage-dependent (2D) cell culture were immunoblotted using the indicated antibodies. (**c**) Expression levels of pChk1 (S296) and total Chk1 were determined by densitometry from (**a**,**b**) above.

We sought to understand how altering the tumour cell growth environment from simple, anchorage-dependent culture on plastic plates to growth as xenografts in immunocompromised mice altered the basal Chk1 activation state. A comparative analysis of Chk1 phosphorylation levels in the same tumour cell lines growing as xenografts in *nu/nu* mice (Fig. 4a) or on plastic (Fig. 4b) identified profound differences in the levels of pChk1 (S296), a marker of Chk1 kinase activity, and pChk1 (S317), an ATR phosphorylation site necessary for Chk1 activation, between the two growth systems. Some cell lines, such as A2058, A2780 and SW480, exhibited a high pChk1 (S296)/Chk1 ratio in both culture systems (Fig. 4c) but the majority of cell lines exhibited greater Chk1 autophosphorylation in anchorage-dependent culture compared to the xenograft. To address potential issues of sample variability, this observation was confirmed in HT29 and Colo205 cells by comparing pChk1 (S296) expression in cell lysates prepared from three different tumour xenografts or plates of cells (Supplementary Fig. S4). In addition to the differences in auto-phosphorylated Chk1 levels, there was little correlation between the levels of pChk1 (S317) or total Chk1 protein between the tumour xenograft and cell line panels (Supplementary Fig. S4). There was a weak correlation between Chk1 phosphorylation on S296 and S317 in the lysates derived from the anchorage-dependent growth (R^2^ = 0.211) but no correlation in the tumour xenograft cell lysates (R^2^ = 0.002) (Supplementary Fig. S4). In the case of A2058 and HT29 cells, growth as multi-cellular tumour spheroids resulted in a DDR protein phosphorylation pattern that more closely resembled the tumour xenograft than cells growing anchorage-dependently.

### Cdc6 expression correlates closely with Chk1 expression in human cancers

**Figure 5:**
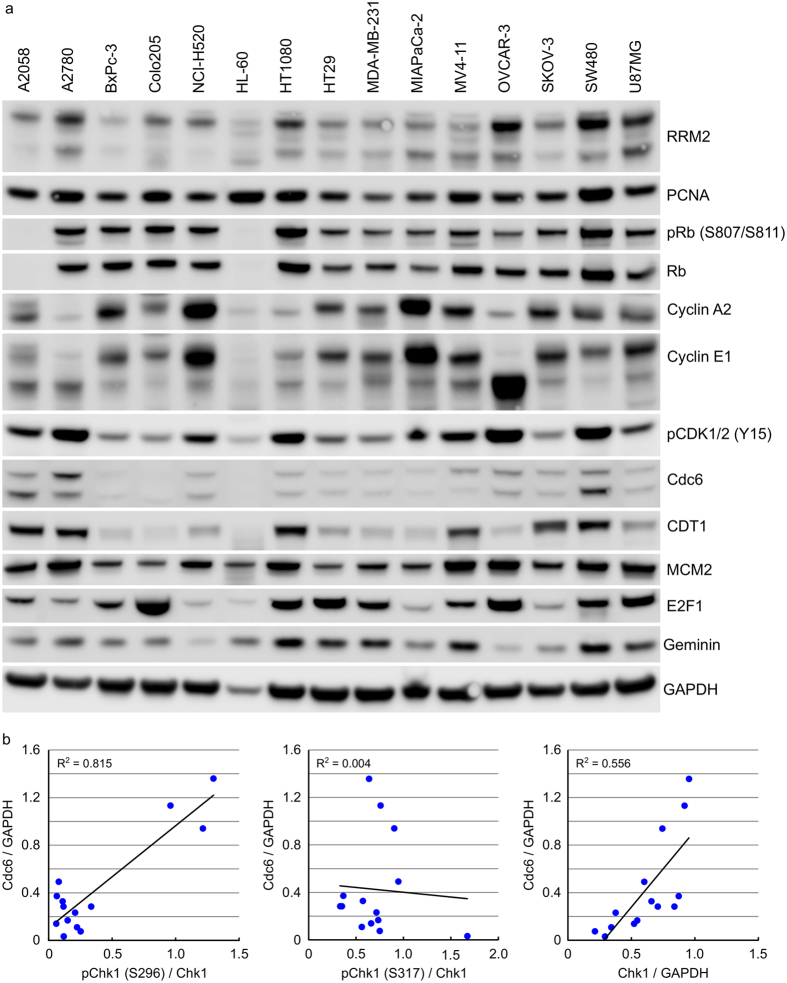
Cdc6 expression correlates closely with Chk1 protein levels and Chk1 auto-phosphorylation in human tumour xenograft samples. (**a**) Cell lysates prepared from cell line derived tumour xenografts were immunoblotted using the indicated antibodies. (**b**) Expression levels of pChk1 (S296), total Chk1 and Cdc6 were determined by densitometry from (**a**) above and Fig. 4a.

**Figure 6:**
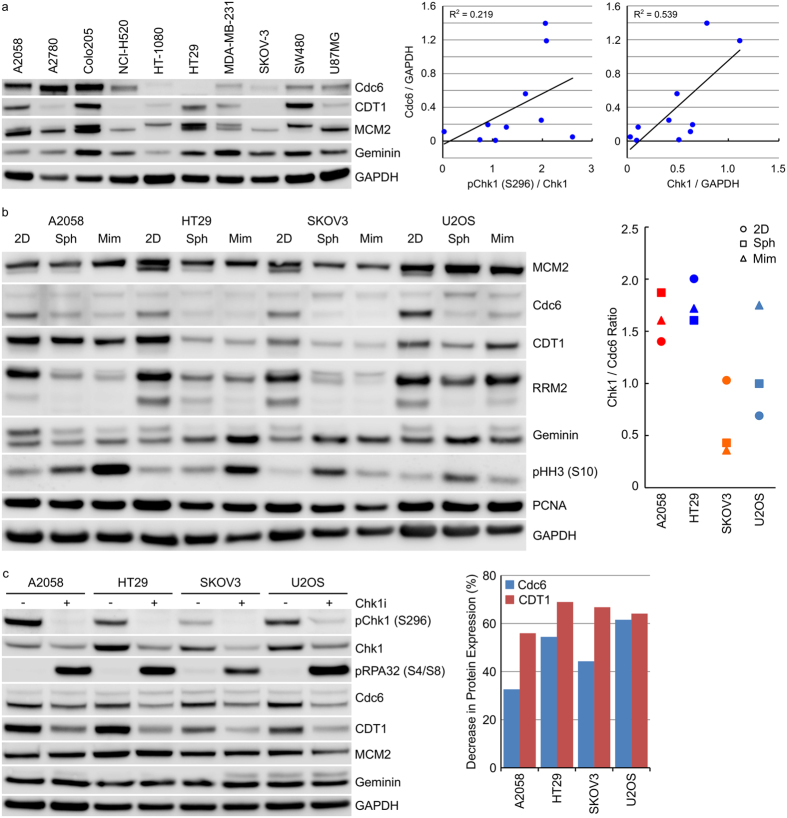
Cdc6 expression correlates closely with Chk1 protein in extracts from human tumour cells. (**a**) Cell lysates from human tumour cell lines growing anchorage-dependently were immunoblotted for the indicated proteins. (**b**) Tumour cell lines were grown anchorage dependently (2D), as multi-cellular tumour spheroids (Sph) for 10 days or in mimetix 3D scaffold (Mim) for 14 days. Cell lysates were immunoblotted with the indicated antibodies. (**c**) Tumour cell lines growing anchorage-dependently were treated with 1 μM Chk1i for 24 hours. Cell lysates were immunoblotted for the indicated proteins. Expression levels of pChk1 (S296), total Chk1, Cdc6 and CDT1 were determined by densitometry and from Figs 1b and 2a.

Replication stress arising through dysregulated DNA replication is an intrinsic threat to tumour cell viability. Chk1 performs a central position in maintaining tumour cell viability via its regulation of replication origin firing, protection of stalled replication forks and activation of repair of collapsed replication forks. Chk1 inhibitors induce DNA damage in S-phase cells^24^,^25^ and reducing the number of actively replicating cells decreases sensitivity to Chk1 inhibitor induced DNA damage and replication stress. Expression levels of a range of proteins involved in DNA replication (see Supplementary Table S1 for details of protein function) was determined in protein extracts from a panel of tumour cell lines growing as xenografts in *nu/nu* mice (Fig. 5a). Expression levels were compared to those already determined for signalling components of the DDR pathway (Fig. 4a and Supplementary Fig. S5). In these tumour xenograft samples, a correlation was observed between Chk1 S296 phosphorylation and Cdc6 (R^2^ = 0.815) and a weaker correlation between total Chk1 protein and Cdc6 (R^2^ = 0.556) but not between pChk1 (S317) and Cdc6 (R^2^ = 0.004) (Fig. 5b). There was no correlation between Chk1 expression and the E2F-regulated gene cyclin A or between Chk1 expression and any of the other cell cycle associated proteins investigated. A similar analysis of tumour cells growing anchorage-dependently revealed a very weak correlation between Chk1 S296 phosphorylation and Cdc6 (R^2^ = 0.219) but a similar correlation between total Chk1 protein and Cdc6 (R^2^ = 0.539) as observed in the xenograft samples (Fig. 6a and Supplementary Fig. S5). No correlation was again observed between pChk1 (S317) and Cdc6 (R^2^ = 0.141, Supplementary Fig. S5). Adapting the tumour cell growth environment from an anchorage-dependent to -independent one resulted in decreased Chk1 protein expression (Fig. 3a) as well as decreased Cdc6, CDT1 and RRM2 protein expression (Fig. 6b). Inhibition of Chk1 with 1 μM Chk1i for 24 hours reduced Cdc6 protein expression levels by 33–62% and CDT1 protein levels by 56–69% (Fig. 6c). There was a weak correlation between Chk1 expression and *in vitro* sensitivity to growth inhibition by Chk1i in the 10 cell lines studied (R^2^ = 0.306) but surprisingly, no correlation between Cdc6 protein levels and *in vitro* sensitivity (R^2^ = 0.022, Supplementary Fig. S6).

**Figure 7:**
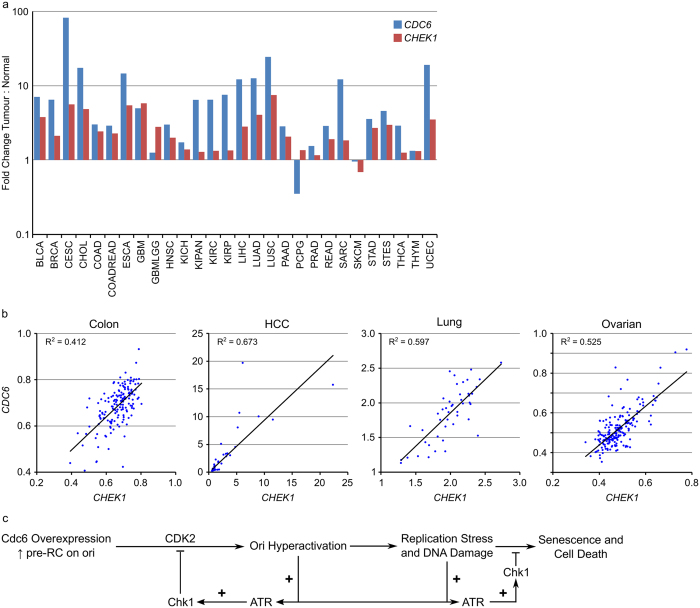
*CDC6* mRNA expression is increased in human cancers and correlates with *CHEK1* mRNA expression. (**a**) Fold changes in *CDC6* or *CHEK1* mRNA expression in human tumours compared to normal tissue controls were determined using the Firehose analysis tool from the Broad Institute TCGA Genome Data Analysis Center. (**b**) The correlation between *CHEK1* (Chk1) and *CDC6* mRNA expression levels was determined from publicly available datasets in colon, HCC, lung and ovarian tumours. (**c**) Model of ATR-Chk1 counterbalance to Cdc6 overexpression-induced origin hyperactivation and replication stress in human cancer.

Fold changes in *CDC6* or *CHEK1* mRNA expression in human tumours compared to normal tissue controls were determined using the Firehose analysis tool from the Broad Institute TCGA Genome Data Analysis Center. *CDC6* mRNA expression was found to be highly upregulated in tumour tissue compared to normal tissue (Fig. 7a and Supplementary Fig. S7) especially in samples derived from cervical carcinoma (CESC), cholangiocarcinoma (CHOL), oesophageal carcinoma (ESCA), liver carcinoma (LIHC), lung adenocarcinoma and squamous carcinoma (LUAD and LUSC), sarcoma (SARC) and uterine corpus endometrial carcinoma (UCEC). In general, elevated expression of *CDC6* mRNA was associated with increased *CHEK1* mRNA expression in the tumour samples compared to normal tissue from the same site. A good correlation between *CDC6* and *CHEK1* mRNA expression levels in colon (R^2^ = 0.412), HCC (R^2^ = 0.673), lung (R^2^ = 0.597) and ovarian (R^2^ = 0.525) but not breast (R^2^ = 0.099) cancer (Fig. 7b and Supplementary Fig. S7) was identified from mRNA expression datasets of patient derived material in the Gene Expression Omnibus (GEO) database. A similar analysis of gene expression data from the 1036 cancer cell lines present in the Cancer Cell Line Encyclopaedia^26^ revealed similar, though less robust, correlations between *CDC6* and *CHEK1* mRNA expression levels in colon (R^2^ = 0.515), CNS (R^2^ = 0.352), liver (R^2^ = 0.325), oesophageal (R^2^ = 0.509), ovarian (R^2^ = 0.240) and pancreatic (R^2^ = 0.311) but not breast (R^2^ = 0.060) or lung (R^2^ = 0.072) cancer (Supplementary Fig. S7).

## Discussion

The major focus of the clinical development of Chk1 inhibitors has been as chemopotentiators of standard-of-care cytotoxic chemotherapy drugs such as gemcitabine, pemetrexed and cisplatin^11^. There is emerging evidence that Chk1 inhibitors may possess anti-tumour efficacy when administered as a single agent. Pre-clinical anti-tumour activity has been observed in a range of cancer models including leukaemia, lymphoma, neuroblastoma, melanoma and breast cancer^12^,^13^,^14^,^16^,^18^,^19^. Tumour cells harbouring high levels of intrinsic replication stress are hypothesised to demonstrate increased dependence on Chk1 for replication and are therefore more sensitive to Chk1 inhibitor induced cell death. This approach is currently being tested in clinical trials with LY2606368^24^ in BRCA1/2 mutation associated breast or ovarian cancer, triple negative breast cancer, high grade serous ovarian cancer, metastatic castrate-resistant prostate cancer, small cell lung cancer, and paediatric solid CNS tumours.

The vast majority of Chk1 inhibitor single-agent pre-clinical studies have utilised established cancer cell lines growing anchorage-dependently. In reality, human tumours exist as complex organs growing in three-dimensions with complex cell-cell interactions and gradients of pH, nutrients and oxygen. We sought to understand how growth *in vitro*, compared to growth *in vivo* affected DDR pathway activation and may predict sensitivity to Chk1 inhibition. We observed clear differences in the phosphorylation state of Chk1 between the same cell line growing anchorage-dependently and as a xenograft in immuno-deficient mice. Several explanations could account for these observed differences: (i) sample preparation results in protein phosphorylation variability, (ii) the antibodies utilised are cross-reactive and recognise both human (tumour) and mouse (stromal) components of the tumour which are inseparable in the xenograft samples, (iii) cell line validation by single tandem repeat (STR) profiling does not capture cell line changes induced by adaptation to *in vivo* growth, or (iv) the tumour microenvironment has profound effects on intrinsic replication stress and DDR pathway activation.

Anchorage-independent growth of tumour cells as multicellular tumour spheroids resulted in a DDR protein phosphorylation pattern that more closely resembled that found in xenograft samples but decreased tumour cell sensitivity to Chk1i. Tumour cell lines grown as multi-cellular tumour spheroids exhibited reduced sensitivity to Chk1 inhibition compared to the same cell line grown anchorage-dependently. Chk1 inhibition reduced biomarkers of Chk1 activity in tumour spheroids suggesting reasonable penetration of Chk1i into the spheroids. In comparison, growth in low serum increased the sensitivity of two of the four cell lines studied. Chk1 inhibitors induce DNA damage during S-phase^24^ and decreasing replication (without inducing DNA damage) decreases the sensitivity of tumour cells in culture to Chk1 inhibitors. Multi-cellular tumour spheroids with their reduced replication rate and nutrient/pH gradients may, therefore, serve as a better model for identifying biomarkers of tumour sensitivity to Chk1 inhibitors than anchorage-dependent culture.

An analysis of replication-associated protein expression identified a correlation between Cdc6 and autophosphorylated or total Chk1 *in vivo* and total Chk1 *in vitro* but not with Chk1 phosphorylated on S317 in either growth scenario. Cdc6 is an essential replication origin licensing factor that, along with ORC, CDT1 and MCM2-7, forms the pre-replication complex (pre-RC) on replication origins during G1- phase^27^,^28^. Cdc6 is essential for DNA replication including the initiation of DNA replication through its ATPase dependent loading of several MCMs at a single origin. Phosphorylation of the pre-RC by CDK2 facilitates the loading of the replicative helicase cofactor Cdc45 and subsequent origin activation. Oncogenic transformation leads to the loss of the controls restricting the onset of S-phase. Activation of ATR and Chk1 protect cells from oncogenic replication stress through cell cycle arrest induction, fork stabilisation and inhibition of cleavage, activation of homologous recombination repair and inhibition of new origin firing^10^. Activation of replication origins is controlled by ATR and Chk1 during a normal S-phase through control of Cdk2^29^,^30^ and Cdc7/Dbf4^31^.

Chk1 and Cdc6 are both regulated by the Rb/E2F pathway^32^,^33^ and the correlation in expression observed could simply be a consequence of cell cycle status. No correlation, however, was observed between Cdc6 or Chk1 expression and E2F1 or the E2F-regulated cyclin A1. Likewise, no correlation in expression was observed between Chk1 or Cdc6 and other proteins involved in the cell cycle. Over-expression of Cdc6 in human cancers leads to origin hyperactivation, uncoordinated origin firing and replication stress. For the cancer cell, persistent replication stress and DNA damage can be deleterious resulting in apoptosis and DNA damage induced senescence^34^. Co-ordinated upregulation and activation of Chk1 to prevent excess origin firing, potentially through the inhibition of Cdk2 and Cdc7, thereby compensating for the effects of Cdc6 overexpression. Inhibition of Chk1 resulted in down-regulation of Cdc6 and CDT1 protein expression suggesting a dynamic equilibrium between Chk1 and Cdc6 balancing the need for origin firing and initiation of DNA synthesis against over activation, replication stress, DNA damage and ultimately cell death. Paradoxically, no correlation between Cdc6 expression levels and sensitivity to Chk1i was observed *in vitro*. Inhibiting Chk1 appears to decrease Cdc6 and CDT1 protein levels thereby decreasing origin hyperactivation but increasing replication stress from active replication factories (Fig. 7c). Further work is needed to dissect how Chk1 and Cdc6 co-operate to balance the need for replication origin firing to complete DNA replication versus excess origin firing-induced replication stress.

## Methods

### Cell lines and cell culture

Cell lines were purchased from the American Type Culture Collection (ATCC), established as a low passage cell bank and then routinely passaged in our laboratory for less than 3 months after resuscitation. These were routinely cultured in media containing 10% FCS and 1% penicillin/streptomycin at 37 °C in a normal humidified atmosphere supplemented with 5% CO_2_. Cells were authenticated by STR profiling (LGC Standards, Teddington UK).

### Spheroid and Mimetix Growth Conditions

Multi-cellular tumour spheroids were grown in ultra-low attachment 96 well or 6 well plates (Corning) as previously described^35^. For mimetix 3D scaffold growth, 1 × 10^5^ cells were plated onto a mimetix 3D scaffold insert (Electrospinning Corporation) in a 6 well plate. Plates were incubated at 37 °C, 5% CO_2_ for 14 days with media replenished approximately every 3 days.

### Compounds

V158411 was from Vernalis Research and prepared as a 20 mM DMSO stock. Compounds were serially diluted in DMSO to 500× or 1000× then to 5× or 10× in complete media before addition to cells to yield a 1× final concentration.

### Antibodies

Antibodies against pChk1 (S296), Cdc6, E2F1, RPA32 and RRM2, and were purchased from Abcam; pCDK1/2 (Y15), CDT1, pChk1 (S317), Chk1, pChk2 (T68), Chk2, Cyclin A2, Cyclin E1, pDNA-PKcs (S2056), DNA-PKcs, GAPDH, Geminin, pH2AX (S139), pHH3 (S10), Ki67, MCM2, PCNA, pRb (S807/S811) and Rb from Cell Signaling Technologies; pRPA32 (S4/S8) from Bethyl Laboratories and pH2AX (S139) (clone JBW301) from Merck Millipore. Antibodies were used at the manufacturer’s recommended dilutions.

### Tumour Samples

Cryogenically powdered tumour samples from human tumour xenograft models were purchased from Charles River Discovery Research Services. Cell lines (to generate xenograft models) were obtained from ATCC or NCI and authenticated by STR profiling. 20–30 mg of tumour was homogenized in RIPA buffer containing protease and phosphatase inhibitor cocktails (Roche) using a Precellys 24 homogeniser and centrifuged twice at 4 °C for 10 minutes at 13 000 rpm. Protein concentration was determined using a BCA kit (Pierce).

### Immunoblotting

Following washing with PBS, cells were lysed in RIPA buffer containing protease and phosphatase inhibitor cocktails (Roche). Protein concentration was determined using a BCA kit (Pierce). Equal amounts of lysate were separated by SDS-PAGE and western blot analysis conducted using the antibodies indicated above. Primary antibodies were detected with HRP-conjugated secondary antibody (Santa Cruz Biotechnology) and detected with Western Lightning (Perkin Elmer) or Immobilon (Millipore) chemiluminescent HRP substrate. Blots were imaged using an LAS 4000 luminescence imager (Fujifilm). Densitometry was determined using Image J software (NIH).

### Single Cell Immunofluorescent Imaging

Single cell immunofluorescent imaging was conducted as previously described^36^. In brief, following compound treatment, cells were fixed in 3.7% paraformaldehyde in PBS at room temperature for 15 minutes, washed with PBS, blocked with 5% normal goat serum in 0.3% Triton X100 in PBS for 60 minutes at room temperature then incubated with primary antibody diluted in antibody dilution buffer (1% BSA, 0.3% Triton X100 in PBS) at 4 °C for 16 hours. Cells were washed with PBS then incubated with an Alexa-labelled secondary antibody (1:500, Life Technologies) and Hoechst 33342 (1 μg/ml) in antibody dilution buffer at room temperature for 60 minutes. Following washing with PBS, cells were imaged with an Operetta high content imaging system (Perkin Elmer) at 10× or 20× magnification and analysed using Harmony software (Perkin Elmer).

### High Content Cell Cycle Analysis

High content cell cycle analysis was conducted essentially as previously described^37^. For DNA only analysis, cells were fixed and permeabilised with 3.7% paraformaldehyde/0.3% Triton X100 in PBS at room temperature for 15 minutes. Cells were washed twice in PBS then stained with Hoechst 33342 (1 μg/ml) in PBS at room temperature for 30 minutes.

For multiparametric cell cycle analysis, cells were labelled with 10 μM EdU for 15 minutes immediately prior to fixation with 3.7% paraformaldehyde in PBS at room temperature for 15 minutes. Cells were washed twice in PBS then twice in 3% BSA in PBS before permeabilisation with 0.5% Triton X100 in PBS for 20 minutes at room temperature. Cells were washed twice with 3% BSA in PBS before incorporated EdU was labelled with an Alexa Click-iT EdU labelling kit (Life Technologies). Following blocking for 30 minutes with 5% normal goat serum in PBS, cells were incubated with an anti-pHH3 (S10) primary antibody diluted in antibody dilution buffer (1% BSA, 0.3% Triton X100 in PBS) at 4 °C for 16 hours. Cells were washed with PBS then incubated with an Alexa-labelled secondary antibody (1:500, Life Technologies) and Hoechst 33342 (1 μg/ml) in antibody dilution buffer at room temperature for 60 minutes. Following washing with PBS, cells were imaged with an Operetta high content imaging system (Perkin Elmer) at 10× magnification and analysed using Harmony software (Perkin Elmer).

### Cell Proliferation Assay

5000 cells per well were seeded in 96-well plates and incubated overnight. Cells were treated with a 10-point titration of compound for 72 or 168 hours. The effect on cell proliferation was determined with sulphorhodamine B (SRB) after fixation with 10% trichloroacetic acid and read on a Victor plate reader (Perkin Elmer). GI_50_ values were calculated in Microsoft EXCEL using an XLFit software add-in (ID Business Solutions).

2000 cells/well were seeded in 96-well round bottomed ultra-low attachment microplates (Corning Costar), centrifuged at 1000 × g for 3 minutes and spheroids formed for 72 hours. Spheroid cell viability was determined using CellTiter-Glo Luminescent Cell Viability Assay (Promega).

### High Content Live Cell Imaging

Cells were seeded in 96 well CellCarrier plates (Perkin Elmer) and allowed to attach for 24 hours before addition of compound. Images were acquired as indicated using the brightfield and digital phase imaging modalities on the Operetta high content imaging system at 10× magnification. Temperature was maintained at 37 °C and CO_2_ at 5% with the live cell chamber module.

Cell confluency was determined from the brightfield images using the Find Texture Regions building block coupled with PhenoLOGIC texture based segmentation in the Harmony software. Cell number was determined by analysis of the digital phase images with the Find Cells building block in Harmony.

### Gene Expression Analysis

The NCBI Gene Expression Omnibus (GEO) (http://www.ncbi.nlm.nih.gov/projects/geo/) was used to compare *CHEK1* and *CDC6* mRNA expression levels in breast (GSE29431), colon (GSE13294), HCC (GSE6764), NSCLC (GSE18842) and ovarian (GSE26712) datasets. Background signal intensity was subtracted and data normalised to the housekeeping gene *HPRT1*. Where more than one probe was associated with a single gene the average expression across the probes for the gene of interest was used.

### Statistical Analysis

Results were analysed using a 2-tailed Student’s t-Test tool within the data analysis package provided by Microsoft Excel.

## Additional Information

**How to cite this article**: Massey, A. J. Tumour growth environment modulates Chk1 signalling pathways and Chk1 inhibitor sensitivity. *Sci. Rep.* **6**, 35874; doi: 10.1038/srep35874 (2016).

## Supplementary Material

Supplementary Information
