# Supplementary material for: Tumour growth environment modulates Chk1 signalling pathways and Chk1 inhibitor sensitivity
Source: Sci Rep. 2016 Oct 24;6:35874. doi: 10.1038/srep35874 (PMC5075878; doi:10.1038/srep35874)
Supplement: Supplementary Information [file srep35874-s1.pdf]

Tumour growth environment modulates Chk1 signalling pathways and sensitivity to Chk1 inhibition

Andrew J Massey

Supplementary Information

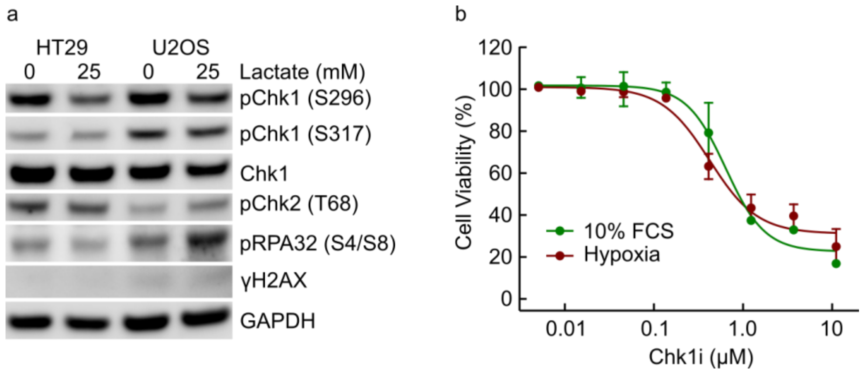

Supplementary Figure S1. Related to Fig. 1.

(a) HT29 or U2OS cells in 10% FCS were exposed to 25 mM sodium lactate for 24 hours. Cell lysates were immunoblotted using the indicated antibodies. (b) HT29 cells growing in 10% FCS/normoxia or 10% FCS/hypoxia were treated with increasing concentrations of Chk1i for 72 hours. Cell viability was determined using SRB (n=3, mean ± SD).

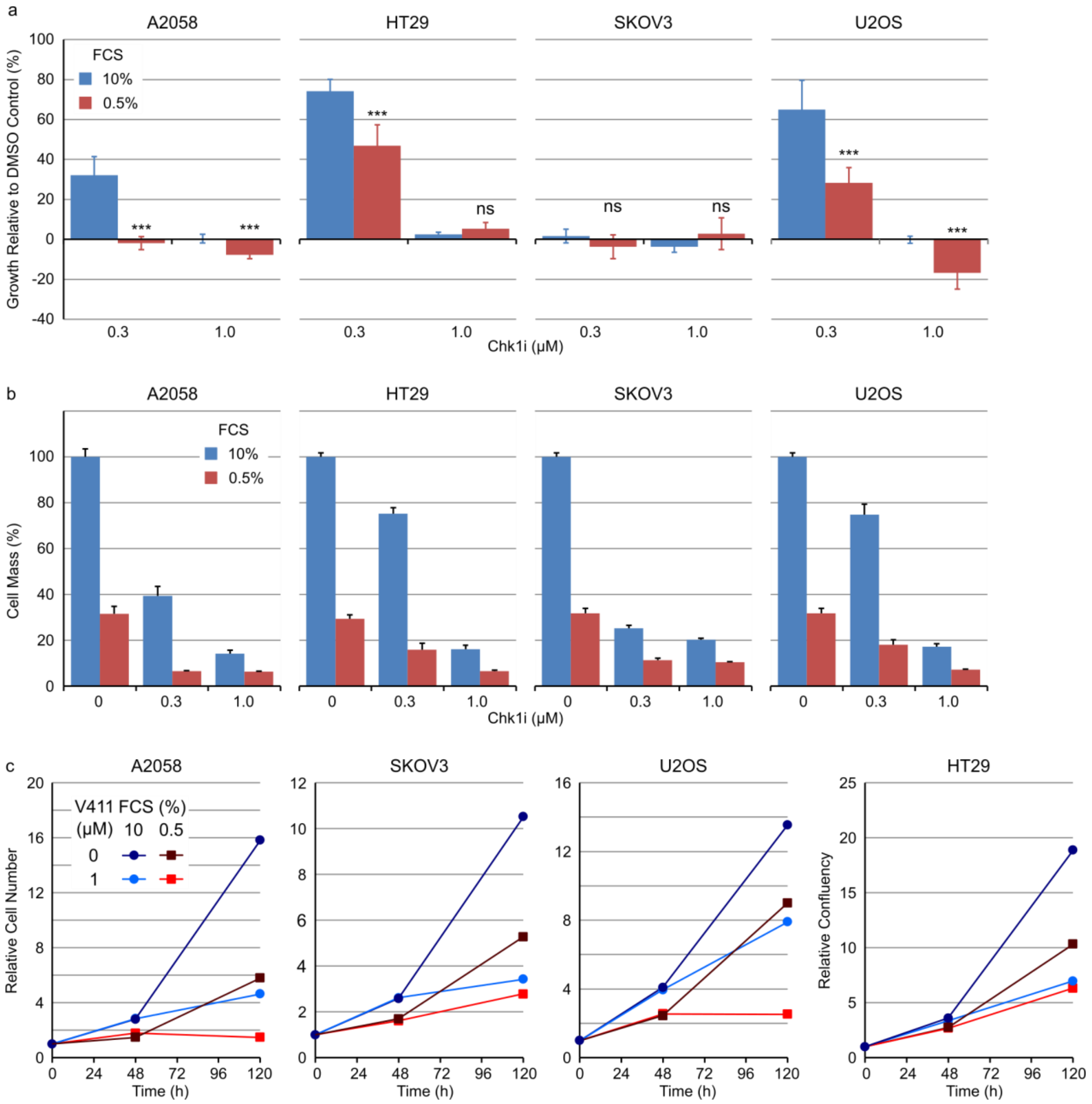

Supplementary Figure S2. Related to Fig. 2.

(a) The percentage changes in growth of cells treated with 0.3 or 1.0 µM Chk1i for 96 hours compared to DMSO control were calculated from Figure 2A. Negative growth indicates a reduction in cell number at 96 hours compared to time 0. Statistical significance was determined using a 2-tailed t-test. ns, not significant; ***, $P < 0.001$. (b) Tumour cell lines growing in 10% or 0.5% FCS were treated with 0 – 1 µM Chk1i for 96 hours. Cell mass was determined by SRB staining (n=6, mean ± SD). (c) Tumour cell lines were grown in 10% or 0.5% FCS for 48 hours then treated with 0 or 1 µM Chk1i (in the same FCS percentage) for a further 72 hours. Cell number or confluency was determined using repeated live cell imaging (n=6, mean).

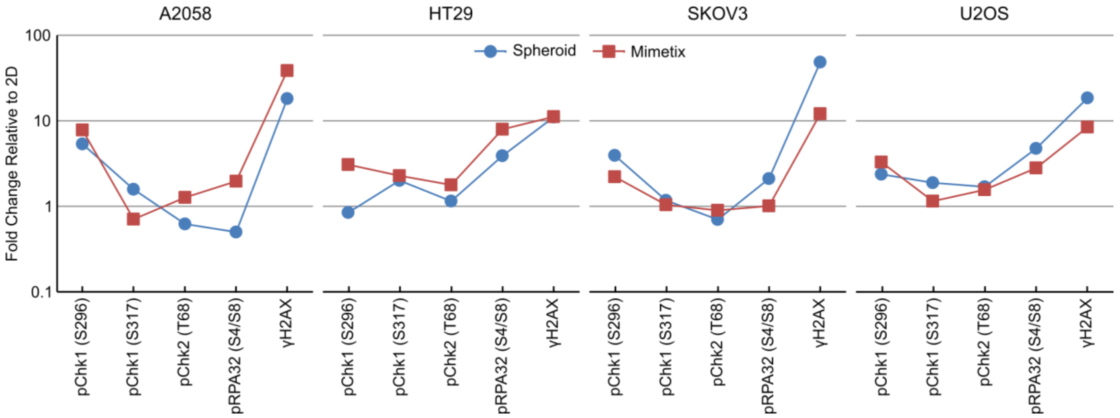

Supplementary Figure S3. Related to Fig. 3.

Protein expression data in figure 3A was quantified by densitometry and the ratios of pChk1 (S296) / Chk1, pChk1 (S317) / Chk1, pChk2 (T68) / Chk2, pRPA32 (S4/S8) / RPA32 and γH2AX / GAPDH calculated. From these, the fold change relative to 2D for cells grown as spheroids or on the mimetix scaffold calculated.

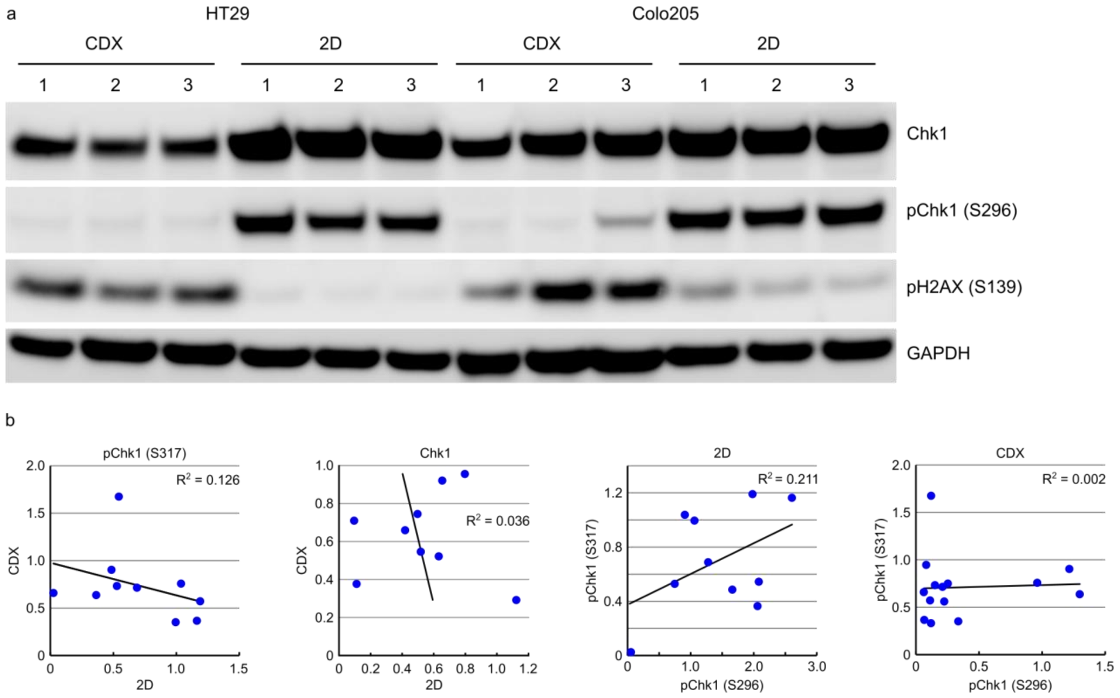

Supplementary Figure S4. Related to Fig. 4.

(a) Cell lysates prepared from HT29 or Colo205 cell line derived tumour xenografts (CDX) or anchorage-dependent cell culture (2D) were immunoblotted using the indicated antibodies. (b) Protein expression levels of pChk1 (S296), pChk1 (S317) and total Chk1 were determined by densitometry from Figure 4 western blots of cell lysates derived from cell lines growing as tumour xenografts (CDX) or anchorage-dependently (2D).

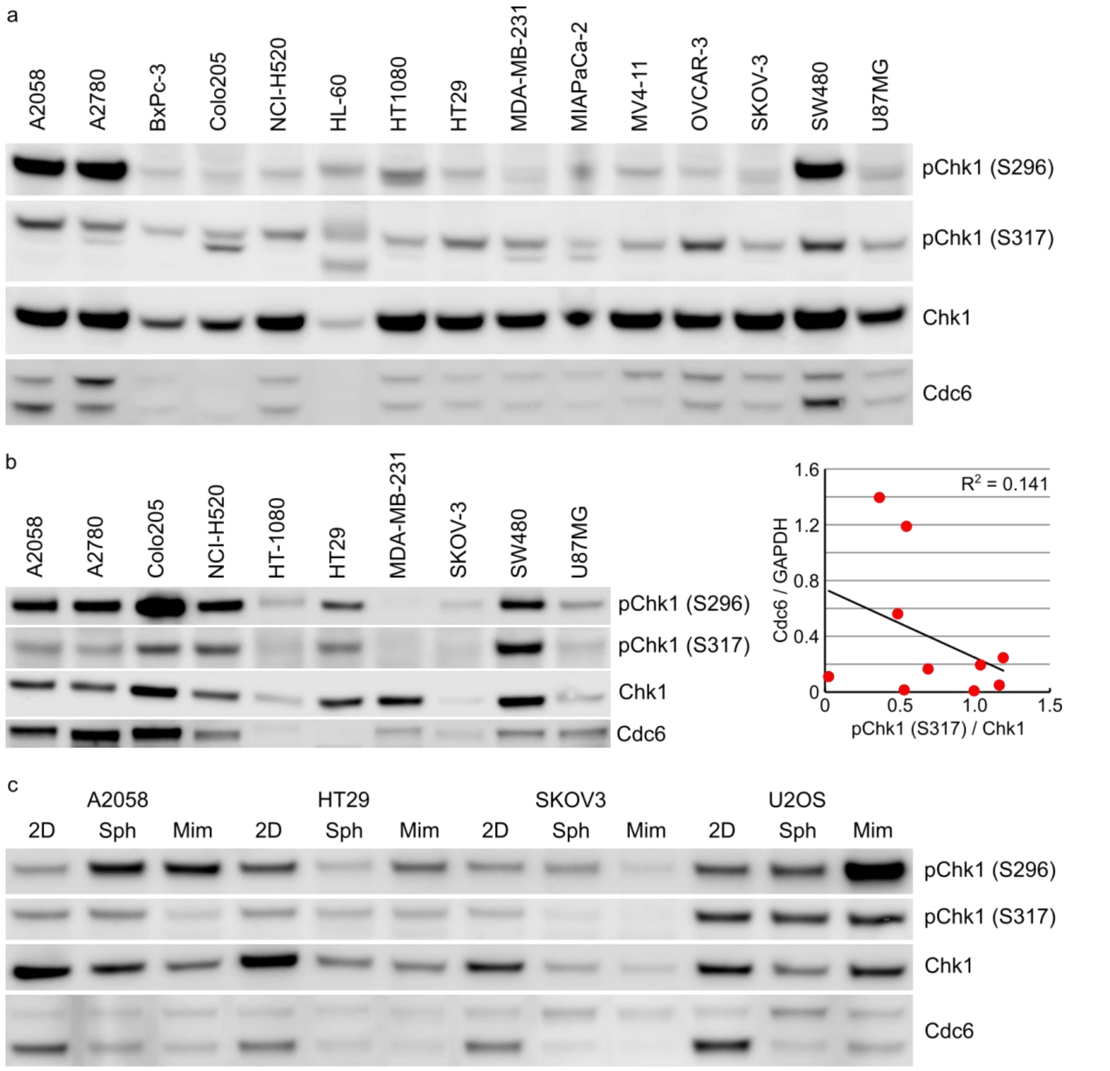

Supplementary Figure S5. Related to Fig. 4, 5 and 6.

Composite images of pChk1 (S296), pChk1 (S317), Chk1 and Cdc6 expression data from (a) Fig. 4a and 5a, (b) Fig. 4b and 6a and (c) Fig. 3a and 6b. Protein expression levels in (b) were determined by densitometry.

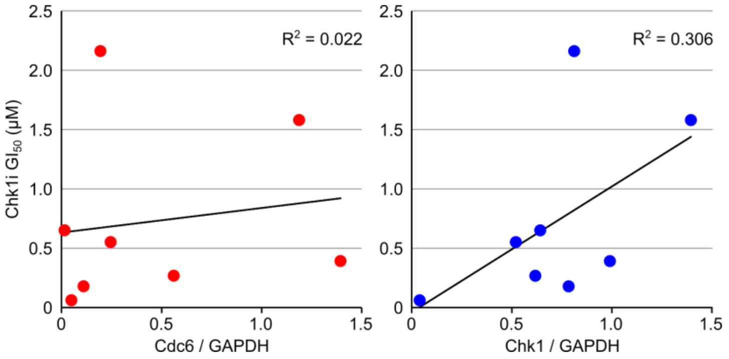

Supplementary Figure S6. Related to Fig. 6.

Expression levels of Cdc6 and Chk1 determined in figure 6A were correlated with the 72 hour Chk1i GI_{50}.

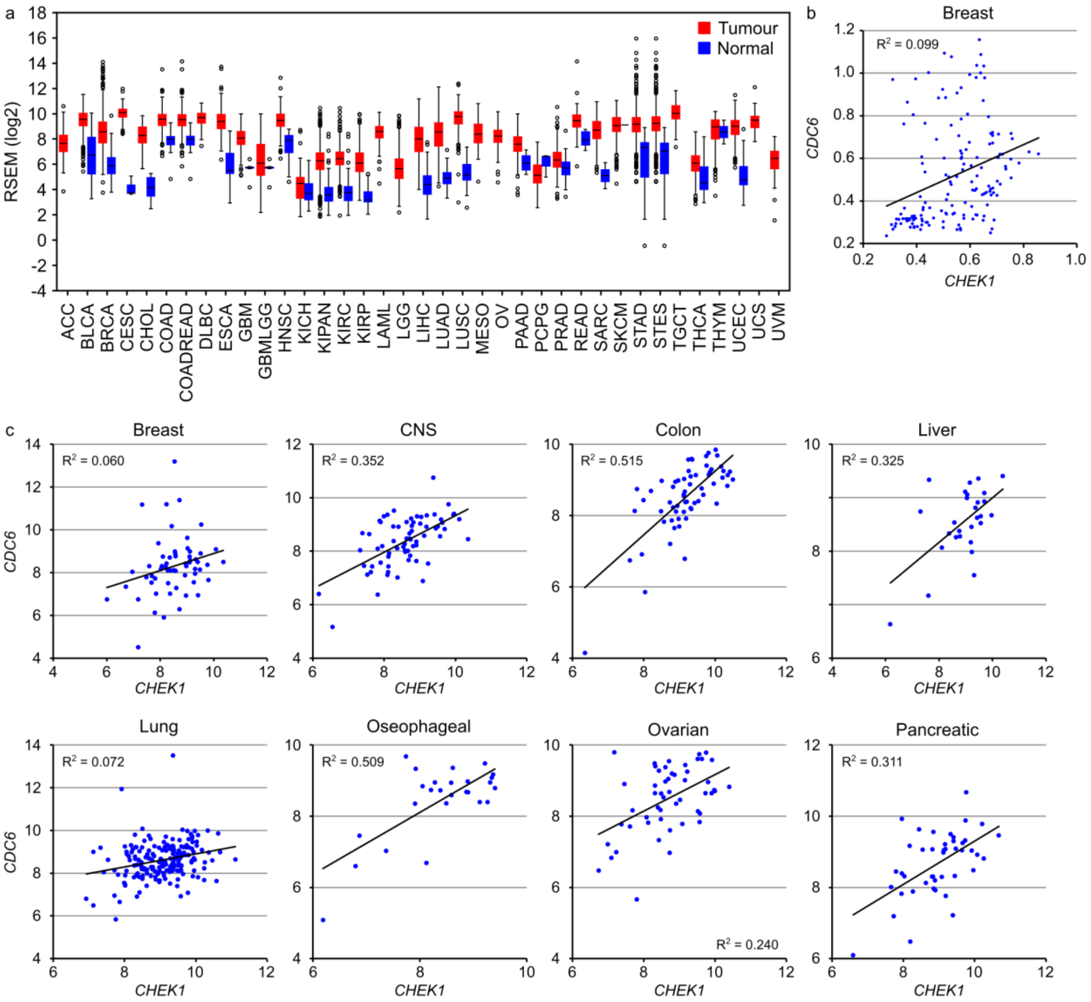

Supplementary Figure S7. Related to Fig. 7.

(a) *CDC6* or *CHEK1* mRNA expression in human tumours and normal tissue controls was determined using the Firehose analysis tool from the Broad Institute TCGA Genome Data Analysis Center. (b) The correlation between *CHEK1* (Chk1) and *CDC6* mRNA expression levels was determined from publicly available datasets in breast tumours. (c) The correlation between *CHEK1* (Chk1) and *CDC6* mRNA expression levels in breast, CNS, colon, liver, lung, oesophageal, ovarian or pancreatic cancer derived cell lines was determined using publically available data from the Broad Institute Cancer Cell Line Encyclopaedia.

Supplementary Table S1. Summary of proteins used in experiments.

Abbrv	Protein Name	Notes
ATM	Ataxia-telangiectasia mutated	STK, mol. sensor of DNA damage predominantly DSB
ATR	ATM- and RAD3-related	STK, mol. sensor of DNA damage predominantly ssDNA
Cdc6	Cell division control protein 6 homolog	Role in initiation of DNA replication and preventing re-replication before mitosis completed
CDK1	Cyclin dependent kinase 1	STK, regulates G2-M transition, P on Y15 by Wee1 (negative regulator of CDK activity)
CDK2	Cyclin dependent kinase	STK, regulates G1-S and S-phase transition, P on Y15 by Wee1 (negative regulator of CDK activity)
CDT1	DNA replication factor Cdt1	Co-operates with Cdc6 to load MCM onto replication origins
Chk1	Checkpoint kinase 1	STK, effector kinase of DRR, cis-autoP on S296, P on S317 and S345 by ATR
Chk2	Checkpoint kinase 2	STK, effector kinase of DDR, P on T68 by ATM
Cyclin A2	Cyclin-A2	Regulates cell cycle transitions. Complex with CDK2 in S-phase and CDK1 in G2/M
Cyclin E1	Cyclin-E1	Regulates cell cycle transitions. Complex with CDK2 in G1 phase, regulates G1-S transition
DNA-PKcs	DNA-dependent protein kinase catalytic subunit	STK, mol. sensor of DNA damage, involved in NHEJ of DSB
E2F1	Transcription factor E2F1	TF of genes whose products are involved in cell cycle regulation or DNA replication
GAPDH	Glyceraldehyde-3-phosphate dehydrogenase	Loading control
Geminin	Geminin	Inhibits MCM incorporation into pre-RC. Degraded during mitosis, re-expressed in S-phase
HH3	Histone H3	P on S10 by Aurora B, marker of mitosis
Ki-67	Antigen KI-67	Expressed in all phases of the cell cycle except G0
MCM2	DNA replication licensing factor MCM2	Subunit of mini-chromosome maintenance complex (MCM 2-7)
PCNA	Proliferating cell nuclear antigen	Auxiliary protein of DNA pol δ; involved in the control of eukaryotic DNA replication
Rb	Retinoblastoma-associated protein	Key regulator of entry into G1, transcription repressor of E2F1 genes, P on S807 / S811 by CDKs
RPA32	Replication protein A, 32kDa Subunit	Subunit of trimeric ssDNA RPA binding protein, P on S4/S8 by DNA-PKcs
RRM2	Ribonucleoside-diphosphate reductase subunit M2	Catalyses biosynthesis of deoxyribonucleotides from ribonucleotides, predominantly S-phase expressed
γH2AX	Histone H2AX	Variant of Histone H2A, P on S139 by DNA-PKcs (also ATR and ATM)

P, phosphorylated; STK, serine / threonine protein kinase; DDR, DNA damage response; mol., molecular; NHEJ, non-homologous end joining; DSB, DNA double strand break; ssDNA, single stranded DNA; CDK, cyclin-dependent kinase; TF, transcription factor
Protein function summarised from UniProt (http://www.uniprot.org/uniprot/)
